# Impulsivity and epilepsy: a bidirectional mendelian randomization study

**DOI:** 10.1186/s42494-024-00181-4

**Published:** 2024-10-15

**Authors:** Tao Chen, Yuqi Liao, Peiwei Hong

**Affiliations:** Department of Neurology, West China School of Public Health and West China Fourth Hospital, Sichuan University, 18th, Section 3, Renmin Road South, Wuhou District, Chengdu, 610041 China

**Keywords:** Mendelian randomization analysis, Epilepsy, Impulsivity, Causal effect

## Abstract

**Background:**

Previous studies have found that patients with epilepsy are more likely to suffer impulsivity. However, the causal relationship between impulsivity and epilepsy is unknown. In this study, we conduct a bidirectional Mendelian randomization (MR) study to explore the causal relationship between impulsivity and epilepsy with recurrent seizure.

**Methods:**

Data of the genome-wide association studies (GWAS) on 14 impulsivity traits and epilepsy were obtained from the GWAS catalog and UK Biobank. Inverse-variance weighted (IVW) and weighted median (WM) methods were utilized for MR estimates. IVW, MR-Egger regression, and MR-pleiotropy residual sum and outlier (MR-PRESSO) methods were used to assess heterogeneity and pleiotropy.

**Results:**

Single-nucleotide polymorphisms (SNPs) related to the lack of perseverance were associated with a decreased risk of epilepsy with recurrent seizures according to the results of IVW (odd ratio [OR] = 0.93, 95% confident interval [CI] = 0.90–0.97, *P* = 0.001) and WM (OR = 0.93, 95%CI = 0.87–0.98, *P* = 0.007). Meanwhile, heterogeneity was not observed with a Cochran Q-derived *P* value of 0.819 for MR egger and a *P* value of 0.808 for IVW. Pleiotropy was not found according to the MR-PRESSO (*P* = 0.273). The other 13 impulsivity traits had no causal effect on epilepsy with recurrent seizures. Meanwhile, SNPs related with epilepsy with recurrent seizures had no causal effect on the 14 impulsivity traits.

**Conclusions:**

This MR study suggests that lack of perseverance may be a protective factor against epilepsy with recurrent seizures. However, epilepsy with recurrent seizures does not affect impulsivity.

## Background

Impulsivity refers to the predisposition toward rapid, unplanned reactions to external or internal stimuli without consideration of the negative consequences of these responses to the impulsive individuals or to others [1]. A previous study of impulsivity measures found three distinct domains, including impulsive choice, impulsive action, and impulsive personality traits measured through self-reported questionnaires [2]. Epilepsy is a chronic neurological disease characterized by sudden abnormal discharges of neurons in the brain, resulting in transient brain dysfunction [3]. Epilepsy with recurrent seizures could result in hippocampal sclerosis [4, 5], and the hippocampus mediates behavior [6]. Furthermore, hippocampal-prefrontal interactions are involved in emotional behaviors [7]. Previous studies found that epilepsy is associated with impulsivity, and impulsivity affects the quality of life of epileptic patients [8–15]. Furthermore, a functional brain magnetic resonance imaging (MRI) study found that the abnormality of anatomical connections, including prefrontal-limbic network, temporo-occipital network, and frontostriatal circuit, might underlie the association between impulsivity and epilepsy [15]. But the causal effect between impulsivity and epilepsy remains unclear.

Fortunately, a recent genome-wide association study (GWAS) revealed the genetic basis of different impulsivity phenotypes [16]. Mendelian randomization (MR) is a method that establishes the causal relationship between different phenotypes by using genetic variants linked to modifiable exposures as proxies for the phenotype [17–19]. In this study, we used bidirectional MR to explore the causal effect between impulsivity and epilepsy with recurrent seizures.

## Methods

A two-sample MR study was performed to explore the causal relationship between impulsivity and epilepsy with recurrent seizures. First, we employed the genetic variants associated with 14 impulsivity traits as instrumental variables (IVs) to determine their causal effect on epilepsy with recurrent seizures. Then, we employed the genetic variants associated with epilepsy with recurrent seizures as IVs to determine its causal effect on impulsivity. All of data analyzed in this study were obtained from public databases. 

### Data source for impulsivity

The most recent impulsivity-associated GWAS study was utilized for the selection of genetic instruments (Table 1) [16]. That study included 1534 participants with European ancestry. The impulsivity included three distinct domains, including impulsive choice, impulsive action, and impulsive personality, which were measured by the Monetary Choice Questionnaire, Go/No-Go task, and the S-UPPS-P scale, respectively [16]. The impulsivity traits included 14 different variables, which are impulsive action factor (IAF), impulsive choice factor (ICF), impulsive personality factor (IPF), impulsive action with No-Go trial accuracy (IA-NGTA), impulsive action with Go trial accuracy (IA-GTA), delayed reward discounting (DRD), delayed reward discounting with small magnitude rewards (DRD-SMR), delayed reward discounting with medium magnitude rewards (DRD-MMR), delayed reward discounting with large magnitude rewards (DRD-LMR), negative urgency, lack of perseverance, lack of premeditation, positive urgency, and sensation seeking [16].

**Table 1:** Sources of GWAS data used in this study

Trait	Consortium	Sample size	Number of cases
Epilepsy, recurrent seizures, convulsions	UK Biobank	400,296	5087
IAF	Deng et al.^a^	1534	NA
ICF	Deng et al.^a^	1534	NA
IPF	Deng et al.^a^	1534	NA
IA-NGTA	Deng et al.^a^	1534	NA
IA-GTA	Deng et al.^a^	1534	NA
DRD	Deng et al.^a^	1534	NA
DRD-SMR	Deng et al.^a^	1534	NA
DRD-MMR	Deng et al.^a^	1534	NA
DRD-LMR	Deng et al.^a^	1534	NA
Negative urgency	Deng et al.^a^	1534	NA
Lack of perseverance	Deng et al.^a^	1534	NA
Lack of premeditation	Deng et al.^a^	1534	NA
Positive urgency	Deng et al.^a^	1534	NA
Sensation seeking	Deng et al.^a^	1534	NA

*GWAS* Genome-wide association study, *NA* Not applicable, *IAF* Impulsive action factor, *ICF* Impulsive choice factor, *IPF* Impulsive personality factor, *IA-NGTA* Impulsive action with No-Go trial accuracy, *IA-GTA* Impulsive action with Go trial accuracy, *DRD* Delayed reward discounting, *DRD-SMR* Delayed reward discounting with small magnitude rewards, *DRD-MMR* Delayed reward discounting with medium magnitude rewards, *DRD-LMR* Delayed reward discounting with large magnitude rewards

^a^means the reference of Deng et al. [16]

### Data source for epilepsy with recurrent seizures

The GWAS data for epilepsy with recurrent seizures were obtained from the UK Biobank (Table 1), which were analyzed by the SAIGE (Scalable and Accurate Implementation of Generalized) mixed model, a method that diminishes the type I error caused by unbalanced case-control phenotypes [20]. The cohort included 5087 epileptic patients with recurrent seizures and 395,209 controls [20].

### Selection of genetic instruments

Single-nucleotide polymorphisms (SNPs) serve as a genetic instrument to indicate the condition of impulsivity or epilepsy with recurrent seizure. The correlation of genetic instruments with the phenotype should meet the following criteria: (1) a GWAS-correlated *P*-value less than 5 × 10^−8^, or a *P*-value less than 5 × 10^−5^ with a *F* statistic > 10 when no SNPs met the tight criteria; and (2) a linkage disequilibrium coefficient *r*^2^ less than 0.001 [17, 21].

### Statistical analyses

We used two methods to determine MR estimates, the inverse-variance weighted (IVW) method and the weighted median (WM) method [17, 18]. Multiple approaches were employed because their underlying assumptions for horizontal pleiotropy varied [22]. The IVW method assumes that all genetic variants are valid IVs, i.e., the genetic instruments can only affect the outcome through exposure and not through any other pathway [22]. The WM method was performed to supplement IVW estimates [22]. Bonferroni-corrected *P* values less than 0.004 were considered as statistical significant [23].

Sensitivity analysis, including bias analysis for pleiotropy and the MR estimates, was performed. Heterogeneity was evaluated by the IVW method and MR-Egger regression, with a Cochran Q-derived *P* < 0.05 indicating significant heterogeneity. Pleiotropy assessment was performed with the MR-pleiotropy residual sum and outlier methods (MR-PRESSO) [19]. Analyses were performed using the packages TwoSampleMR (version 0.5.6) and MendelR (version 2.1.2) in R software (version 4.0).

## Results

All SNPs selected as IVs had a GWAS-correlated *P* value of no more than 5 × 10^−5^ and a *F* statistics more than 10. No SNPs fulfilled the criterion of *P* < 5 × 10^−8^ (Table 2).

**Table 2:** Number of instrumental variables and associated phenotypic variance

Exposure	Outcome	Number of SNPs	*R* ^2^	Mean F (Min, Max)
IAF	EWRS	48	0.50	16.25 (13.86, 22.99)
ICF	EWRS	36	0.41	17.60 (15.17, 21.26)
IPF	EWRS	35	0.32	14.33 (12.12, 18.22)
IA-NGTA	EWRS	45	0.55	18.56 (15.88, 25.65)
IA-GTA	EWRS	57	0.67	17.64 (15.27, 22.79)
DRD	EWRS	40	0.48	18.52 (16.33, 22.06)
DRD-SMR	EWRS	34	0.39	17.77 (15.51, 22.74)
DRD-MMR	EWRS	40	0.45	17.35 (15.17, 22.54)
DRD-LMR	EWRS	41	0.48	17.96 (15.50, 23.76)
Negative urgency	EWRS	34	0.42	19.20 (16.78, 24.46)
Lack of perseverance	EWRS	41	0.49	18.52 (16.20, 23.75)
Lack of premeditation	EWRS	40	0.49	19.17 (15.77, 26.44)
Positive urgency	EWRS	40	0.49	18.90 (15.98, 24.81)
Sensation seeking	EWRS	35	0.44	19.33 (16.81, 25.23)
EWRS	IAF	8	0.03	1443.27 (1356.41, 1569.39)
EWRS	ICF	13	0.05	1462.62 (1369.72, 1669.24)
EWRS	IPF	13	0.05	1444.14 (1363.59, 1634.59)
EWRS	IA-NGTA	7	0.03	1483.92 (1363.59, 1613.57)
EWRS	IA-GTA	9	0.03	1529.20 (1383.33, 2010.72)
EWRS	DRD	10	0.04	1470.89 (1392.86, 1669.24)
EWRS	DRD-SMR	11	0.04	1537.79 (1369.72, 2052.16)
EWRS	DRD-MMR	10	0.04	1537.36 (1369.72, 2052.16)
EWRS	DRD-LMR	11	0.04	1519.80 (1392.86, 2052.16)
EWRS	Negative urgency	12	0.04	1490.17 (1363.59, 1930.68)
EWRS	Lack of perseverance	11	0.04	1442.50 (1363.59, 1582.68)
EWRS	Lack of premeditation	4	0.01	1390.28 (1363.59, 1434.94)
EWRS	Positive urgency	12	0.04	1494.70 (1363.59, 1634.59)
EWRS	Sensation seeking	11	0.04	1500.58 (1363.59, 1779.39)

*IAF* Impulsive action factor, *ICF* Impulsive choice factor, *IPF* Impulsive personality factor, *IA-NGTA* Impulsive action with No-Go trial accuracy, *IA-GTA* Impulsive action with Go trial accuracy, *DRD* Delayed reward discounting, *DRD-SMR* Delayed reward discounting with small magnitude rewards, *DRD-MMR* Delayed reward discounting with medium magnitude rewards, *DRD-LMR* Delayed reward discounting with large magnitude rewards, *EWRS* Epilepsy with recurrent seizure

### Causal effect of impulsivity on epilepsy with recurrent seizures

There were 48, 36, 35, 45, 57, 40, 34, 40, 41, 34, 41, 40, 40 and 35 independent SNPs associated with IAF, ICF, IPF, IA-NGTA, IA-GTA, DRD, DRD-SMR, DRD-MMR, DRD-LMR, negative urgency, lack of perseverance, lack of premeditation, positive urgency, and sensation seeking, respectively. The *R*^2^, which stands for phenotypic variance explained by the SNPs, and *F* statistics of every IV, are shown in Table 2.

**Fig. 1:**
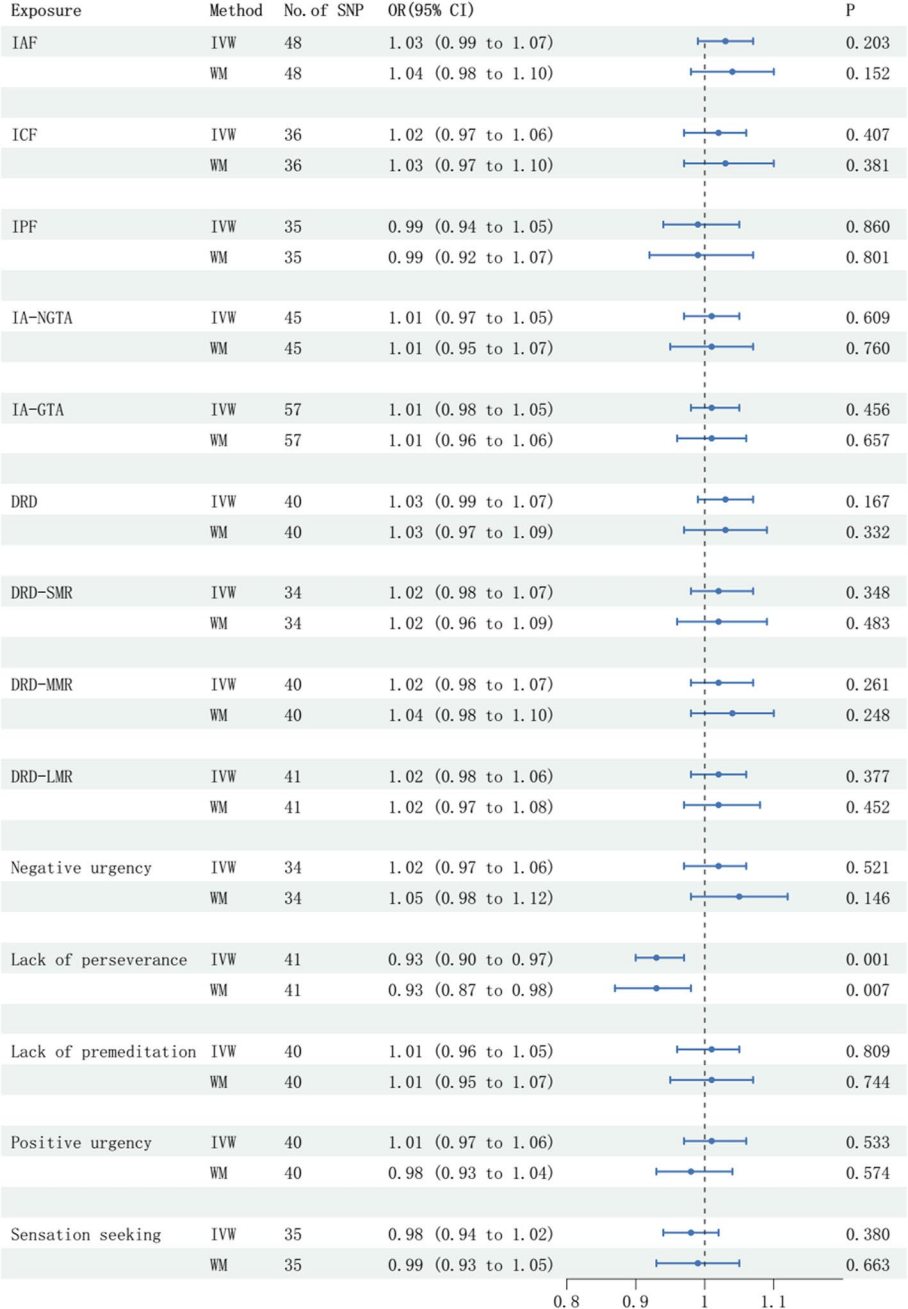
The forest plot showing the causal effect of 14 impulsivity traits on epilepsy with recurrent seizures, estimated with the IVW and WM methods. Lack of perseverance was shown to reduce the risk of epilepsy with recurrent seizures IVW: inverse variance weighted; WM: weight medium; OR: odd ratio; CI: confidence interval; IAF: impulsive action factor; ICF: impulsive choice factor; IPF: impulsive personality factor; IA-NGTA: impulsive action with No-Go trial accuracy; IA-GTA: impulsive action with Go trial accuracy; DRD: delayed reward discounting; DRD-SMR: delayed reward discounting with small magnitude rewards; DRD-MMR: delayed reward discounting with medium magnitude rewards; DRD-LMR: delayed reward discounting with large magnitude rewards

The SNPs related with lack of perseverance were associated with a decreased risk of epilepsy with recurrent seizures according to the IVW (odd ratio [OR] = 0.93, 95% confident interval [CI] = 0.90–0.97, *P* = 0.001) and WM (OR = 0.93, 95%CI = 0.87–0.98, *P* = 0.007) methods (Fig. 1). Meanwhile, heterogeneity was not observed with a Cochran Q-derived *P* value of 0.819 for MR egger and a *P* value of 0.808 for IVW. Pleiotropy was not found according to the MR-PRESSO (*P* = 0.273) (Table 3). We also found that there was no significant causal effect of other SNPs related with impulsivity traits on the risk of epilepsy with recurrent seizures (Fig. 1).

**Table 3:** Heterogeneity and pleiotropy

	Heterogeneity		Pleiotropy
	MR Egger	IVW	MR-PRESSO
Exposure			
IAF	0.443	0.477	0.669
ICF	0.555	0.577	0.471
IPF	0.108	0.116	0.459
IA-NGTA	0.252	0.273	0.551
IA-GTA	0.220	0.169	0.097
DRD	0.603	0.576	0.221
DRD-SMR	0.991	0.993	0.703
DRD-MMR	0.392	0.432	0.742
DRD-LMR	0.993	0.982	0.090
Negative urgency	0.297	0.262	0.190
Lack of perseverance	0.819	0.808	0.273
Lack of premeditation	0.380	0.176	0.014
Positive urgency	0.469	0.430	0.176
Sensation seeking	0.616	0.647	0.572
Outcome			
IAF	1.000	1.000	0.905
ICF	1.000	1.000	0.948
IPF	1.000	1.000	0.896
IA-NGTA	1.000	1.000	0.964
IA-GTA	1.000	1.000	0.902
DRD	1.000	1.000	0.931
DRD-SMR	1.000	1.000	0.841
DRD-MMR	1.000	1.000	0.946
DRD-LMR	1.000	1.000	0.914
Negative urgency	1.000	1.000	0.878
Lack of perseverance	1.000	1.000	0.949
Lack of premeditation	0.990	0.999	0.986
Positive urgency	1.000	1.000	0.798
Sensation seeking	1.000	1.000	0.945

*IVW* Inverse variance weighted, *MR-PRESSO* MR-Pleiotropy Residual Sum and Outlier methods, *IAF* Impulsive action factor, *ICF* Impulsive choice factor, *IPF* Impulsive personality factor, *IA-NGTA* Impulsive action with No-Go trial accuracy, *IA-GTA* Impulsive action with Go trial accuracy, *DRD* Delayed reward discounting, *DRD-SMR* Delayed reward discounting with small magnitude rewards, *DRD-MMR* Delayed reward discounting with medium magnitude rewards, *DRD-LMR* Delayed reward discounting with large magnitude rewards

### Causal effect of epilepsy with recurrent seizures on impulsivity traits

**Fig. 2:**
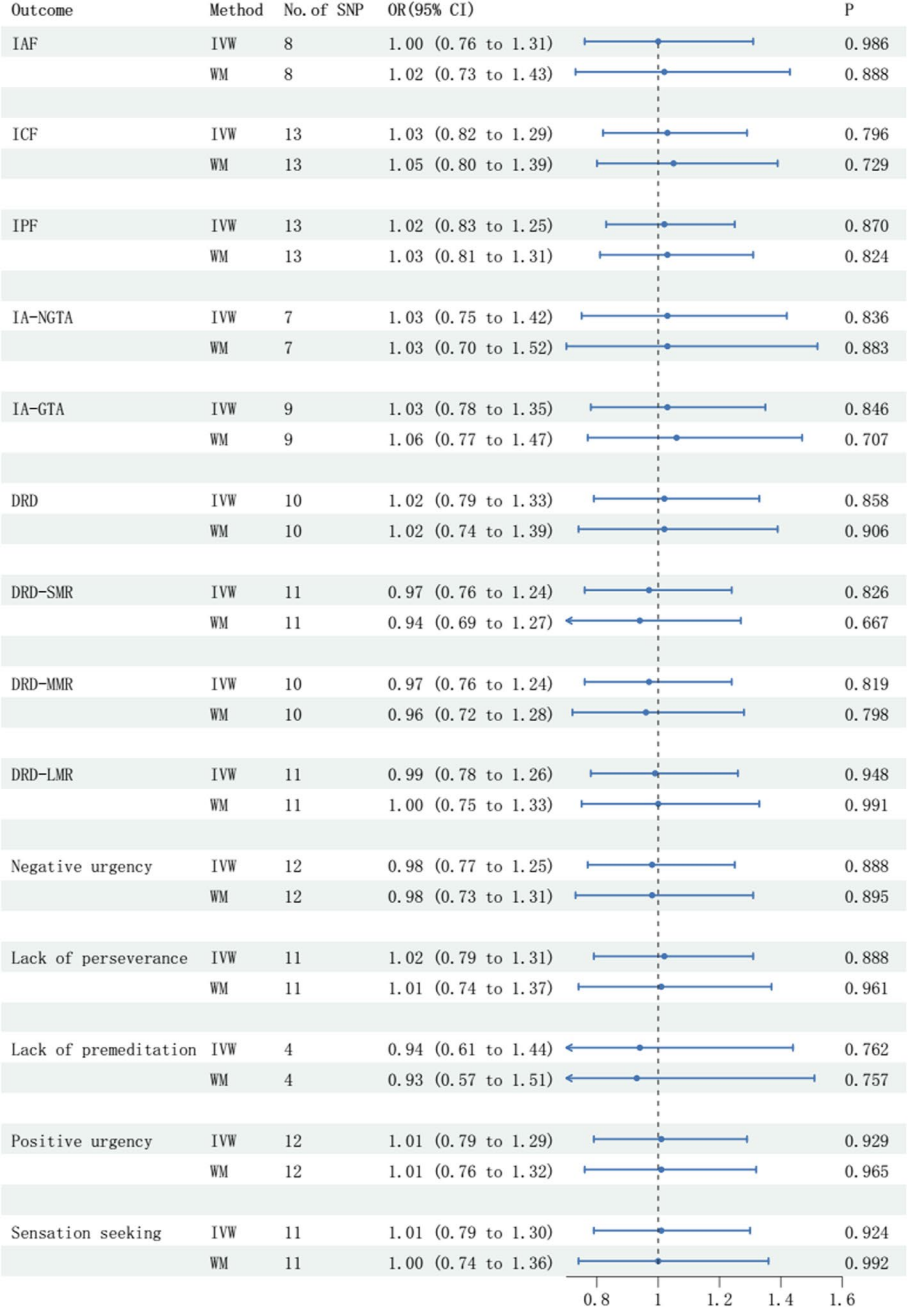
Forest plot showing the causal effect of epilepsy with recurrent seizures on 14 impulsivity traits estimated with the IVW and WM methods. Results showed that there were no causal effects of epilepsy with recurrent seizures on the 14 impulsivity traits. IVW: inverse variance weighted; WM: weight medium; OR: odd ratio; CI: confidence interval; IAF: impulsive action factor; ICF: impulsive choice factor; IPF: impulsive personality factor; IA-NGTA: impulsive action with No-Go trial accuracy; IA-GTA: impulsive action with Go trial accuracy; DRD: delayed reward discounting; DRD-SMR: delayed reward discounting with small magnitude rewards; DRD-MMR: delayed reward discounting with medium magnitude rewards; DRD-LMR: delayed reward discounting with large magnitude rewards

The number of SNPs associated with epilepsy with recurrent seizures, selected as IVs, were different after harmonizing with different impulsivity traits (Fig. 2). The *R*^2^ and *F* statistic of every IV are shown in Table 2. There were no causal effects of SNPs related with epilepsy with recurrent seizures on 14 different impulsivity traits (Fig. 2). In addition, no heterogeneity or pleiotropy was observed (Table 3).

## Discussion

Our study was the first MR study exploring the bidirectional causal effects between impulsivity and epilepsy with recurrent seizures. The results showed that lack of perseverance is a protective factor against epilepsy with recurrent seizures. Meanwhile, epilepsy with recurrent seizures did not affect the 14 impulsivity traits.

Previous clinical studies have found that impulsivity is associated with epilepsy. Patients with juvenile myoclonic epilepsy show increased impulsivity [9, 10, 12, 14, 24, 25]. Patients with idiopathic generalized epilepsy or frontal lobe epilepsy are more likely to have high impulsivity [15]. Furthermore, impulsivity is associated with the severity of epileptic seizures [26]. However, in a previous study, patients with temporal lobe epilepsy showed no differences in impulsivity, as compared with healthy controls [15]. Our MR analysis was the first to analyze the relationship between three distinct domains of impulsivity and epilepsy with recurrent seizures. We found that epilepsy could not induce impulsivity. However, we found that lack of perseverance is a protective factor against epilepsy with recurrent seizures. The mechanism of this causal relationship is unknown, which may involve the frontal lobe networks [15, 27].

Perseverance, also known as grit, is defined as the passion for long-term goals. Perseverance is an important noncognitive trait [28]. In our study, lack of perseverance was utilized for measuring the impulsive personality traits [16]. A study found that the volume of nucleus accumbens is associated with interindividual differences in perseverance [29]. In another study with 217 healthy adolescent students, Wang et al. found that perseverance is negatively associated with the fractional amplitude of low-frequency fluctuations in the right dorsomedial prefrontal cortex [30]. These results demonstrated that the prefrontal cortex and the basal ganglia are the neural basis of perseverance. Furthermore, Myers et al. found that perseverance was associated with the connectivity between dorsal and ventral striatum and the dorsal anterior cingulate cortex [31]. These neural basis and neural network of perseverance might affect epileptic seizures through hippocampal-prefrontal interactions, which need to be verified in the future [7].

This study had some limitations. First, the data used in this study were all from European ancestors, so the conclusions should be applied with caution to other ethnic populations. Second, the mechanisms of the causal effect between lack of perseverance and epilepsy are unclear. Brain functional imaging can help clarify the mechanisms. Third, the results might be affected by age, gender, and ethnicity. Future studies with participant stratification according to these confounders are needed. Finally, our study did not classify the types of epilepsy, which might cause false-negative results.

## Conclusions

In conclusion, the MR study provides evidence that the lack of perseverance may be a protective factor against epilepsy with recurrent seizures. However, epilepsy with recurrent seizures do not affect impulsivity.

## Data Availability

The data analyzed in this study are available on the UK Biobank (https://pheweb.org/UKB-SAIGE/) and GWAS catalog (https://www.ebi.ac.uk/gwas/).

## Abbreviations

CI: Confidence interval
DRD: Delayed reward discounting
DRD-LMR: Delayed reward discounting with large magnitude rewards
DRD-MMR: Delayed reward discounting with medium magnitude rewards
DRD-SMR: Delayed reward discounting with small magnitude rewards
GWAS: Genome-wide association studies
IAF: Impulsive action factor
IA-GTA: Impulsive action with Go trial accuracy
IA-NGTA: Impulsive action with No-Go trial accuracy
ICF: Impulsive choice factor
IPF: Impulsive personality factor
IVs: Instrumental variables
IVW: Inverse variance weighted
MR: Mendelian randomization
MRI: Magnetic resonance imaging
MR-PRESSO: MR-pleiotropy residual sum and outlier
OR: Odd ratio
SNPs: Single-nucleotide polymorphisms
WM: Weight medium
